# Type 2 Diabetes Mellitus and Increased Risk for Malaria Infection

**DOI:** 10.3201/eid1610.100399

**Published:** 2010-10

**Authors:** Ina Danquah, George Bedu-Addo, Frank P. Mockenhaupt

**Affiliations:** Author affiliations: Institute of Tropical Medicine and International Health, Berlin, Germany (I. Danquah, F.P. Mockenhaupt);; Kwame Nkrumah University of Science and Technology, Kumasi, Ghana (G. Bedu-Addo)

**Keywords:** Malaria, vector-borne infections, risk factors, Plasmodium, diabetes mellitus, Ghana, parasites, dispatch

## Abstract

A case–control study of 1,466 urban adults in Ghana found that patients with type 2 diabetes mellitus had a 46% increased risk for infection with *Plasmodium falciparum*. Increase in diabetes mellitus prevalence may put more persons at risk for malaria infection.

In sub-Saharan Africa, infectious diseases remain the predominant cause of illness and death. *Plasmodium falciparum* malaria alone causes an estimated 1 million deaths annually (*1*). At the same time, sub-Saharan Africa faces the world’s highest increase in type 2 diabetes mellitus; adaptation to Western lifestyles and genetic predispositions may accelerate this trend (*2**,**3*). A decade ago, type 2 diabetes mellitus prevalence in urban Ghana was 6.3% (*4*). By 2030, ≈20 million affected persons may live in sub-Saharan Africa (*2*). Type 2 diabetes mellitus increases susceptibility to common infections (*5*). In sub-Saharan Africa, the emerging co-occurrence of type 2 diabetes mellitus and tropical infectious diseases thus may have substantial implications. We describe prevalence of malaria infection in adults with and without type 2 diabetes mellitus residing in Kumasi, Ghana. Malaria transmission in Kumasi is low but patchy; mosquito breeding sites also occur in urban agricultural areas (*6*).

## The Study

A case–control study of risk factors for type 2 diabetes and hypertension was conducted from August 2007 through June 2008 at Komfo-Anokye Teaching Hospital, Kumasi, Ghana. The patients’ clinical and biochemical signs and symptoms were secondary objectives (I. Danquah et al., unpub. data). The study protocol was approved by the Ethics Committee, University of Science and Technology, Kumasi, and participants gave informed written consent.

Patients attending the diabetes (n = 495) or hypertension center (n = 451) were recruited. These patients promoted participation as preliminary (i.e., to be confirmed) controls to community members, neighbors, and friends (n = 222). Further preliminary controls were recruited from the outpatient department (n = 150) and among hospital staff (n = 148).

Participants were told to fast, abstain from alcohol and nicotine use, and avoid stressful and physical activities beginning at 10pm the day before examination. On the day of examination, participants were asked about medical history and socioeconomic background, underwent physical examination, and provided venous blood and urine samples for laboratory testing.

Fasting plasma glucose (hereafter referred to as glucose concentration; fluoride plasma 4°C) and hemoglobin (Hb) concentrations were measured (Glucose-201^+^, B-Hemoglobin; HemoCue, Angelhom, Sweden). Irrespective of symptoms, malaria parasites were counted per 500 leukocytes on Giemsa-stained thick blood films. *Plasmodium* infection and species were ascertained by PCR that included positive and negative controls (*7*).

Patients with type 2 diabetes mellitus were defined as those receiving documented treatment with antidiabetes medication or having a glucose concentration >7 mmol/L (*8*); patients with hypertension were defined as those receiving documented antihypertension treatment or having mean blood pressure >140/90 mm Hg for 3 measurements (*9*). Controls had neither condition.

Between-group comparisons were performed by the Mann-Whitney U, χ^2^, and Fisher exact tests. Logistic regression produced adjusted odds ratios (aORs), and 95% confidence intervals (CIs).

Of the 1,466 study participants, 675 (46%) had type 2 diabetes (Table 1). Among these, 655 (97.0%) received antidiabetes treatment, but 317 (47.0%) had increased glucose concentration (>7 mmol/L). The 414 patients with hypertension but not diabetes and 377 controls with neither illness were grouped despite differences, e.g., in age and socio-economic parameters (data not shown); however, glucose concentration was similar for the two groups (mean 4.51 vs. 4.56 mmol/L; p = 0.53).

**Table 1:** Demographic and clinical characteristics of 1,466 urban residents of Kumasi, Ghana, 2007–2008*

Characteristics	Persons with type 2 diabetes mellitus, n = 675	Persons without diabetes, n = 791	p value
Age, y, mean (range)	54.7 (18–92)	47.1 (18–100)	<0.0001
Male gender	171 (25.3)	182 (23.0)	0.299
Wealth score <25th percentile†	265 (39.6)	271 (34.3)	0.044
Illiteracy	308 (45.8)	206 (26.1)	<0.0001
Formal education, none	240 (35.7)	130 (16.5)	<0.0001
Crowded living condition‡	177 (26.7)	120 (15.3)	<0.0001
Smoking, current or quit	49 (7.3)	35 (4.4)	0.024
Akan ethnicity	592 (87.8)	685 (86.6)	0.480
Residence			
Kumasi metropolitan area	476 (70.8)	603 (76.2)	
Kumasi suburbs	174 (25.9)	162 (20.5)	
Els3where§	22 (3.3)	26 (3.3)	0.048
Occupation			
Public servant	44 (6.5)	194 (24.6)	
Trader	198 (29.5)	190 (24.1)	
Farmer	65 (9.7)	48 (6.1)	
Unemployed	248 (36.9)	138 (17.5)	
Other¶	117 (17.4)	218 (27.7)	<0.0001
FPG, mmol/L, mean (range)	8.3 (1.3–37.1)	4.5 (2.9–7.0)	<0.0001
Hemoglobin, g/dL, mean (range)	12.9 (5.8–19.1)	13.6 (4.9–19.1)	<0.0001
Fever, >37.5°C	2 (0.3)	4 (0.5)	0.693
History of fever, preceding week	95 (14.1)	93 (11.8)	0.182
Respiratory tract infection	5 (0.7)	11 (1.4)	0.232
Urinary tract infection#	14 (2.1)	7 (0.9)	0.076
*Plasmodium* spp. infection, by microscopy	5 (0.7)	8 (1.0)	0.582
Parasite density, per µL, median (range)	1,160 (160–2,480)	860 (80–4,960)	0.770
*Plasmodium* spp. infection, by PCR			
*Plasmodium* spp.	117 (17.4)	89 (11.3)	0.001
* P. falciparum*	108 (16.0)	81 (10.3)	0.001
* P. malariae*	14 (2.1)	9 (1.1)	0.205
* P. ovale*	8 (1.2)	7 (0.9)	0.611

*Values are no. (%) unless otherwise indicated. p values were calculated by Mann-Whitney U test or Fisher exact test, as applicable. FPG, fasting plasma glucose concentration. †<25th percentile of a calculated index of 11 markers of wealth: electricity, pipe-borne water, radio, fan, cupboard, television, bicycle, motorbike, refrigerator, car/truck/tractor, cattle. ‡>75th percentile of the number of persons living in the household. §Hinterland and environs. ¶Includes casual laborer, artisan, and others. #By nitrite-positive urine dipstick test (Combur 10, Roche Diagnostics, Mannheim, Germany).

According to microscopic examination, 13 (0.9%) of all participants had malaria parasites at low density (median 880/µL, range 80–4,960/µL). Reexamination by PCR showed that 206 (14.1%) were infected with *Plasmodium* spp., largely *P. falciparum* (189, 12.9%). Infected persons were afebrile, but mean hemoglobin was reduced (–0.4 g/dL; p = 0.004).

More *Plasmodium* spp. infections were observed in persons with type 2 diabetes mellitus than in those without the disease (Table 1); most infections were caused by *P. falciparum* (16% vs. 10%; p = 0.001). This difference was not attributable to recent antimalarial medication (7 persons with type 2 diabetes mellitus vs. 13 persons without type 2 diabetes mellitus; p = 0.32), and, notably, 74/524 (14.1%) of the patients with type 2 diabetes mellitus who took metformin-based drugs were infected compared with 34/131 (26.0%) of those who did not (p = 0.01). Among controls and patients with hypertension, the *P. falciparum* prevalence was similar (35/377, 9.3% for controls; 46/411, 11.2% for patients with hypertension; p = 0.38), and in each case, it was comparatively higher among patients with type 2 diabetes mellitus (p = 0.003 for controls; p = 0.03 for patients with hypertension).

Several factors that differed between persons with and those without diabetes mellitus (Table 1) were associated with *P. falciparum* infection (Table 2). However, age-adjusted multivariate analysis confirmed that the odds of *P. falciparum* infection in patients with type 2 diabetes mellitus were increased (aOR 1.46; Table 2). This risk increase was still discernible in the same model comparing patients with type 2 diabetes mellitus with controls (aOR 1.68, 95% CI 1.06–2.65; p = 0.027) or patients with hypertension (aOR 1.38, 95% CI 0.94–2.02; p = 0.096), or when separating into metropolitan area (aOR 1.67, 95% CI 1.12–2.48; p = 0.01) and other residence (aOR 1.32, 95% CI 0.76–2.29; p = 0.33).

**Table 2:** Univariate and multivariate associations with *Plasmodium falciparum* infection, Kumasi, Ghana, 2007–2008*

Parameter	Total no. patients	*P. falciparum* infection, no. (%)	Univariate analysis			Multivariate analysis	
			OR (95% CI)	p value		aOR (95% CI)	p value
Diabetes mellitus type 2							
No	791	81 (10.3)	1				
Yes	675	108 (16.0)	1.67 (1.22–2.27)	0.001		1.46 (1.06–2.03)	0.021
Gender							
F	1,113	124 (11.2)	1				
M	353	65 (18.5)	1.80 (1.29–2.50)	<0.0001		2.13 (1.50–3.03)	<0.0001
Wealth score							
>25th percentile	923	94 (10.2)					
<25th percentile †	536	94 (17.6)	1.88 (1.38–2.56)	<0.0001		1.76 (1.27–2.42)	0.001
Literacy							
Able to read	947	103 (10.9)	1				
Unable to read	514	85 (16.6)	1.63 (1.20–2.23)	0.002		1.59 (1.11–2.28)	0.011
Formal education							
Any	1,091	126 (11.6)	1				
None	370	62 (16.8)	1.54 (1.11–2.15)	0.010			
Living condition							
Uncrowded	1,147	133 (11.6)	1				
Crowded‡	297	52 (17.5)	1.61 (1.14–2.29)	0.007			
Smoking							
Never	1,380	171 (12.4)	1				
Current or quit	84	18 (21.4)	1.92 (1.11–3.32)	0.019			
Ethnicity							
Akan	1,277	156 (12.3)	1				
Others	188	33 (17.6)	1.52 (1.01–2.30)	0.045			
Residence							
Kumasi metropolitan	1,079	121 (11.2)	1				
Kumasi outskirts	336	64 (19.2)	1.87 (1.34–2.61)	<0.0001			
Elsewhere §	48	4 (8.3)	0.72 (0.25–2.03)	0.533			
Occupation							
Public servant	238	17 (7.1)	1				
Trader	388	50 (12.9)	1.92 (1.08–3.42)	0.026			
Farmer	113	34 (30.6)	5.74 (3.04–10.86)	<0.0001			
Other¶	335	38 (11.3)	1.66 (0.92–3.02)	0.095			
Unemployed	386	49 (12.8)	1.90 (1.07–3.39)	0.029			

*OR, odds ratio; CI, confidence interval; aOR, adjusted odds ratio. Age and gender were a priori included in the multivariate model. Further variables for inclusion in the model were identified by factor analysis excluding multicollinear parameters (1: retained diabetes, excluded occupation; 2: retained literacy, excluded education, smoking; 3: retained wealth, excluded living condition, ethnicity). The same model results from a logistic regression analysis initially including all above listed parameters, and then removing in a stepwise backward fashion all factors not associated with *P. falciparum* infection in multivariate analysis (p > 0.05). Inserting any of the excluded variables back into the model did not change the aOR of patients with type 2 diabetes mellitus by >7% each, suggesting the absence of substantial confounding. Leaving all parameters in the model yielded an aOR for patients with type 2 diabetes mellitus of 1.36 (95% CI, 0.98–1.90; p = 0.07). Alternatively, propensity score adjustment of that analysis, i.e. reducing covariates into a single variable, produced aOR = 1.41 (95% CI, 1.02–1.95; p = 0.04). †<25th percentile of a calculated index of 11 markers of wealth. ‡Crowded living condition, >75th percentile of the number of persons living in the household, i.e., n>8. §Hinterland and environs. ¶Includes casual labourer, artisan, and others.

According to the multivariate model, exchanging type 2 diabetes mellitus with glucose concentration showed that each mmol/L increase in blood glucose increased the risk for *P. falciparum* infection by 5% (aOR 1.05, 95% CI 1.02–1.09; p = 0.002). Among patients with type 2 diabetes mellitus, a stepwise approach identified 8.6 mmol/L glucose concentration as the significant threshold of risk increase (aOR 1.63, 95% CI 1.07–2.48; p = 0.02).

## Conclusions

This study provides evidence for increased risk for *P. falciparum* infection in patients with type 2 diabetes mellitus (Table 2). Most infections were detected by PCR exclusively, and all were asymptomatic.

Submicroscopic and asymptomatic *P. falciparum* infections are common in areas where malaria is endemic. In adults, PCR may identify up to 50% of infections, although only a few infections are diagnosed by microscopy (*10*). These submicroscopic infections tend to increase in areas of low endemicity and with patient age (*10**)*.

An increased risk for *P. falciparum* infection in persons with diabetes mellitus might become clinically relevant (and microscopically detectable) under several conditions. The impact of semi-immunity on controlling parasitemia may weaken with advancing type 2 diabetes mellitus and immune dysfunction (*5*), as suggested by the observed risk increase with increasing glucose concentration. Conversely, children who lack semi-immunity but have more severe type 1 diabetes mellitus may be particularly prone to malaria. Such vulnerability is also conceivable for women with gestational diabetes whose immune systems are relatively naive with regard to pregnancy-specific *P. falciparum* (*11*). Moreover, low-level infections in patients with type 2 diabetes mellitus may constitute an unrecognized infectious reservoir in areas where malaria is endemic (*10*). The lowered *P. falciparum* prevalence under metformin medication accords with the biguanides’ antimalarial efficacy (*12*).

Our data stem from a study that was not designed to assess influences on *P. falciparum* infection in a heterogeneous population. Multivariate analysis cannot exclude unmeasured confounders, and association does not mean causality. As a limitation, factors influencing infection were not specifically identified during recruitment and thus were not included in analysis. Also, despite adjusting for proxy indicators, e.g., wealth, exposure to infection might still have differed between the study groups, considering the patchy malaria transmission in Kumasi (*6*). Nonetheless, increased odds of *P. falciparum* in patients with type 2 diabetes mellitus were found after stratification by subgroups or residence. Ultimate corroboration would need a prospective, longitudinal study controlling for exposure (possibly monitored by serologic markers of transmission).

Although the actual reasons for the increase of *P. falciparum* infection are unclear, the risk increase with rising glucose concentration is a sign of biologic plausibility. Such risk could result from impaired defense against liver and/or blood-stage parasites and from prolonged persistence. In type 2 diabetes mellitus, decreased T cell–mediated immunity but limited impact on humoral responses are discussed (*5*). Mechanistically, increased glucose availability may feed *P. falciparum* growth as seen in vitro (*13*). Also, patients with diabetes might receive more infectious mosquito bites: olfactory signals mediate mosquito attraction (*14*), and these, including expiration, are subtly altered in patients with type 2 diabetes mellitus (*15*).

The rapid proliferation of type 2 diabetes mellitus in sub-Saharan Africa may put an increasing number of persons at risk for *Plasmodium* infection and malaria. Thus, the magnitude of both diabetes mellitus and malaria in sub-Saharan Africa warrants further investigation into the relevance and causes of our finding
